# Advances in Potassium Silicate-Induced Drought Tolerance in Tropical Tree Seedlings: Effects on Morphological Traits, Physiological Responses, and Biochemical Regulation

**DOI:** 10.3390/plants14243760

**Published:** 2025-12-10

**Authors:** Sylvia Henintsoa Nomenaharinaivo, Dario Donno, Lorenzo Rosso, Giovanni Gamba, Harilala Andriamaniraka, Gabriele Beccaro

**Affiliations:** 1 Dipartimento di Scienze Agrarie, Forestali e Alimentari, Università degli Studi di Torino, 10095 Grugliasco, Italy; dario.donno@unito.it (D.D.); lorenzo.rosso@unito.it (L.R.); giovanni.gamba@unito.it (G.G.); gabriele.beccaro@unito.it (G.B.); 2 Mention Agriculture Tropicale et Développement Durable, Ecole Supérieure des Sciences Agronomiques, Université d’Antananarivo, BP 175, Antananarivo 101, Madagascar; jharilala@gmail.com; 3 Scuola Istituto Universitario di Studi Superiori Pavia, Piazza della Vittoria n.15, 27100 Pavia, Italy

**Keywords:** *Canarium madagascariense*, abiotic stress, water stress adaptation, silicon-based biostimulant, biochemical defense, phytohormones regulation

## Abstract

Water stress is among the most important abiotic constraints affecting forest ecosystem functioning and regeneration, a phenomenon expected to intensify with climate change. It impacts photosynthesis, growth, and seedling survival, therefore threatening biodiversity and accelerating forest degradation. The use of silicon-based biostimulants has emerged as a way of mitigating the effects of water stress by improving water status and stimulating mechanical and biochemical defense. However, its effectiveness on forest tree species remains poorly explored. This study examines how potassium silicate (PS) alleviates the effects of drought on *Canarium madagascariense*, with the aim of improving our understanding of the resilience mechanisms of tropical forest species. To do this, an experiment with 135 two-year-old *C. madagascariense* saplings has been conducted, testing three irrigation levels in combination with the addition of potassium silicate (PS) at concentrations of 5 and 10 mM, via foliar spraying and soil application. Morphometric and physiological parameters were monitored, followed by the biochemical profiling of the induced responses. Linear mixed models were computed to assess the effects of the different factors on the different growth performance, physiological functioning parameters over time, and ANOVA was used for evaluating the punctual data on the biochemical compounds. Drought had a significant impact on the morphological and physiological behaviour of the seedlings. However, the application of PS modified the drought-induced changes, even at a low concentration of 5 mM. Biochemical defenses were also improved further with PS application. Hormone profiling revealed a predominance of auxins, while abscisic acid was lower in the water stress treatments under drought. Therefore, using PS could support the production of robust seedlings that are more tolerant of, and adaptive to, the challenges of climate change, making restoration more efficient.

## 1. Introduction

Water stress is one of the most significant abiotic factors affecting the functioning of forest ecosystems and the services they provide [1]. Due to climate change, as CO_2_ emissions and global temperatures continue to rise, changes to rainfall distribution and patterns and distribution affect the frequency, the intensity, and the duration of meteorological drought [2,3,4]. Water scarcity can significantly alter biological processes in forests, impairing the physiology of species and disrupting the structure and composition of forest ecosystems, as well as their distribution of vegetation. This favors drought-tolerant species at the expense of diversity [4,5]. Moreover, the frequency of drought periods may further accelerate the existing deforestation patterns and diversity loss, particularly in highly fragmented forests with local endemism, which are less resilient [2,3,6]. At the species level, drought can lead to changes in morphological traits, productivity, the survival rate, and reproduction. Particularly, the limited establishment and survival of seedlings due to water stress has a strong impact on species reproduction and, therefore, on forest natural regeneration and population maintenance [6,7,8]. Moreover, the implications of drought open the door to other biotic or abiotic stressors affecting the plant [6].

Depending on the extent and intensity of the stress, plants adopt different strategies in response to drought stress. These strategies vary between species and ecosystems. Globally, these strategies can be categorised as avoidance, tolerance, or escape. Avoidance mechanisms involve modulating plant functions, particularly physiological and morphological processes, to reduce water consumption and enhance uptake in response to low availability. Drought tolerance involves initiating defence mechanisms, whether physiological or biochemical, including gene and hormonal regulation, to mitigate negative effects and prevent fatal outcomes. Escaping drought stress corresponds to shortening the life cycle [9,10]. As these responses differ from one species to another, it is essential to understand how these mechanisms are coordinated in each species in order to assess its adaptive capacity and predict its future evolutionary patterns.

The use of silicon has been well documented as a means of enhancing plant growth and development, as well as regulating resistance to abiotic stress [11,12,13]. Although silicon is the second most abundant element in the Earth’s crust, it is not an essential nutrient for plants. Nevertheless, it plays a pivotal role in modulating their response to drought stress. It improves root development and structure, regulates plant water status, and influences the factors that determine the relationship between plants and water. This ensures efficient access to, uptake of, and transport of water [14]. Furthermore, silicon contributes to structural reinforcement to prevent water loss [15,16]. It has been demonstrated that foliar spraying enhances silicon assimilation and avoids the immobilization issues encountered with soil administration [17]. The role of silicon in drought stress responses has been well explored in many crop plants, such as cereals (rice, wheat, and maize) [18,19,20], as well as horticultural crops such as cucumber, daisy, chestnut, peach [12,21,22,23]. However, the plant’s response may vary depending on the form and concentration of silicon administered, and on its ability to absorb and accumulate the element. The potential of silicon-based biostimulants to alleviate drought stress in forest tree species has not yet been widely explored, as few studies have addressed this issue [24,25]. However, these species are constantly exposed to drought episodes throughout their lifespan, making them particularly vulnerable to this phenomenon. Tropical forest species with a narrow ecological niche have been shown to be particularly susceptible to water stress [26,27]. Understanding how endemic species are affected by water stress, and how they adapt their behavior, can improve our knowledge of how forests adapt to climatic variability. This knowledge can also inform the development of conservation strategies for these species, as well as innovative nursery techniques for producing robust seedlings for forest restoration in the face of current challenges.

The *Canarium madagascariense* is a species with a very specific habitat: the tropical rainforests of Madagascar, where it is native but now endangered of extinction [28,29]. It plays a pivotal role in maintaining the structure of the forest and the food chain for wildlife. It is also of economic and social interest due to its timber and medicinal properties. The multiple uses of this species exacerbate conservation concerns, mainly due to inappropriate harvesting methods [30], the existing deforestation and climatic variability. A previous study suggested that the species was well adapted to water limitations. Nevertheless, these adaptive responses need to be investigated further, as does the potential management practice of using potassium silicate (PS) to improve resilience.

The present study aims to investigate the role of potassium silicate in alleviating the effects of water stress in *C. madagascariense* when administered via foliar spraying and soil addition. Specifically, the study will evaluate how different concentrations of PS modulate morphological traits, physiological functions, and biochemical processes under drought stress conditions, with the aim of understanding the mechanisms by which PS improves seedling tolerance. Ultimately, the outcomes of this study will advance knowledge of silicon-mediated tolerance in woody species and determine the most effective PS application practices.

## 2. Results

### 2.1. Temperature and Humidity Conditions

**Figure 1:**
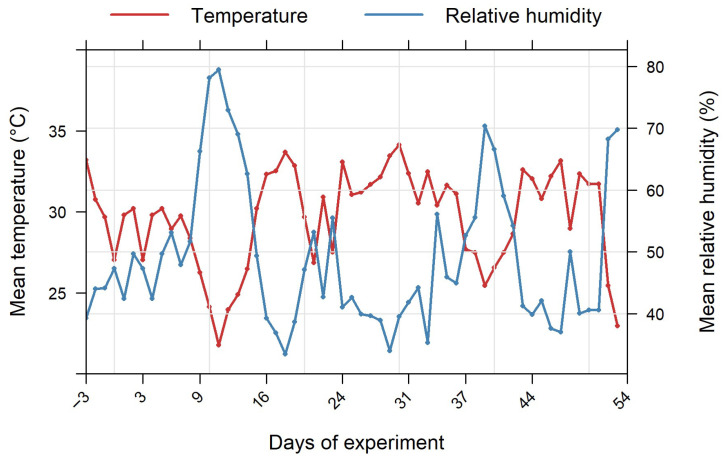
Greenhouse microclimatic conditions during the experiment.

The temperature ranged from 21 °C to 34 °C, while the relative humidity ranged from 33% to 79%. These two variables evolved in inverse proportion (Figure 1). The lowest peak of temperature corresponds to the highest peak in humidity, as air water holding capacity depends on the temperature. Overall, temperatures are high throughout the experiment. Indeed, the period of November-December of the experiment corresponds to the hot and humid season in the locality.

### 2.2. Morphometric Measurements

#### 2.2.1. Height

The PS did not significantly affect the height of the seedlings (df = 2, *p* = 0.34). A likelihood-ratio test confirmed that the best model for the data only includes the irrigation and measurement dates (MD) as fixed factors. Moreover, the ANOVA type III two-way interaction was significant (df = 2, *p* < 0.001), meaning that the evolution over time of height growth differs between irrigation treatments. Under control irrigation conditions (C), plant height increased faster over time (estimate = 1.37, SE = 0.06, t-value = 20.57, *p* < 0.001). At lower irrigation levels, height growth slowed down proportionally to the reduction in irrigation (estimate = 0.72, SE = 0.09, t-value = −6.90, *p* < 0.001 for moderate stress M; estimate = 0.64, SE = 0.09, t-value = −7.80, *p* < 0.001 for severe stress S).

The examination of the random effect revealed that seedling individuals accounted for meaningful variability in the data (SD = 1.15), which was higher than the variance observed in residuals (SD = 1.00). This means that height growth varied within individuals, but variation between individuals was even more significant.

**Figure 2:**
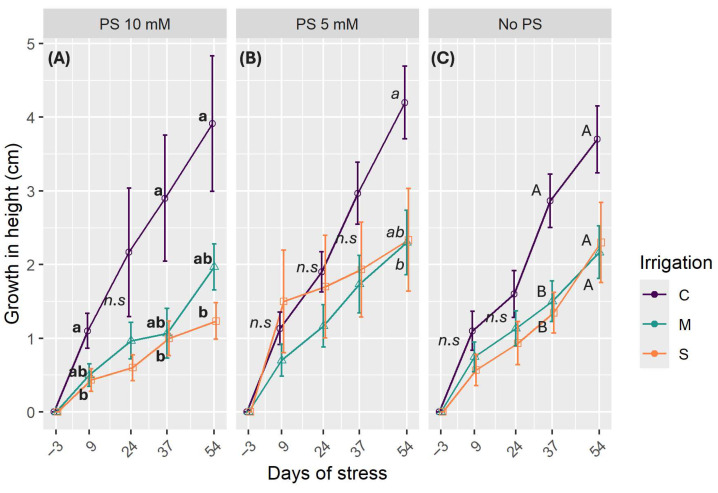
Height growth by PS concentrations: (**A**) Addition of 10 mM PS; (**B**) Addition of 10 mM PS; (**C**) No PS used. C denotes control irrigation treatment (100% Field Capacity); M denotes moderate stress treatment (50% FC); S denotes severe stress treatment (25% FC). Data are mean ± standard error (n = 15). Lowercase bold and italic letters indicate significant differences between irrigation treatments with PS at 10 and 5 mM, respectively, while uppercase letters indicate differences without PS (*p* < 0.05). Error bars represent standard error. n.s.: non-significant differences.

The following graphics (Figure 2) illustrate how the height of *C. madagascariense* seedlings changes over a two-week period in response to different levels of irrigation and PS. Overall, plant height growth is only affected by the level of water in the soil. The growth trend remains consistent across different PS concentrations, except for a slight improvement in control treatment (C) growth with 10 mM PS, where the growth trend is almost linear (Figure 2A). Differentiation between the irrigation treatments emerged slightly earlier than in the other groups. Water deficit treatments result in decreased height growth compared to optimal irrigation conditions. As the experiment progressed, the difference in height between control and stressed plants became more significant. At the end of the experiment, the growth rate of seedlings in the control irrigation treatment was two-fold higher than in stress treatments, which had similar values. By the end of the experiment, the final height growth under optimal conditions (C) was 3.91 ± 0.91 cm, 4.20 ± 0.49 cm, and 3.70 ± 0.49 cm when using 10 mM, 5 mM, and no PS, respectively. Under moderate stress conditions (M), the respective values were 1.97 ± 0.33 cm, 2.30 ± 0.43 cm, and 2.16 ± 0.35 cm. Under severe stress conditions (S), the respective values were 1.23 ± 0.24 cm, 2.33 ± 0.69 cm, and 2.30 ± 0.54 cm (see Figure 2A–C).

#### 2.2.2. Diameter

The model for stem diameter data was fitted with only irrigation and measurement dates (MD) as fixed factors, whereas random factors corresponded to block and plant id. The use of PS did not have a significant effect on the data on diameter (df = 2, *p* = 0.53). The best-fitted model did not include the use of PS, as it was identical to the model with only irrigation and MD and their interaction (χ^2^ = 6.74, *p* = 0.87), which meant that the use of PS did not affect the data. The two-way interaction between irrigation levels and DM had a significant effect on stem diameter (df = 2, *p* < 0.001). The diameter increases over time, but the rate of this increase depends on irrigation treatments. Compared to plants under optimal irrigation conditions (C), those under moderate stress (M) showed a smaller increase in diameter over time (estimate = −0.09, standard error (SE) = 0.02, t-value = −3.22, *p* = 0.001). The increase was even lower in severely stressed plants (S) (estimate = −0.16, SE = 0.02, t-value = −5.70, *p* < 0.001).

The model accounted for variation between plants by including their identity. The variance related to individual plants (SD = 1.35) was higher than the residual variance (SD = 0.30), indicating that the variation between individuals was greater than the variation within individuals.

**Figure 3:**
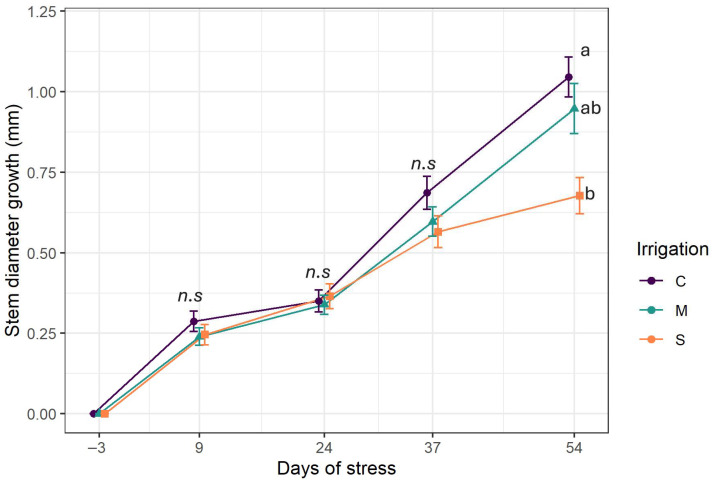
Stem growth evolution. C: control irrigation (100% FC); M: moderate stress (50% FC); S: severe stress (25% FC). Data are mean ± SE (n = 15). Different lower-case letters indicate significant differences between the treatments (*p* < 0.05). n.s.: non-significant differences.

The time series (Figure 3) illustrates the growth of stem diameter for each level of irrigation throughout the experiment. Stem growth followed an upward trend despite the drought onset. During most of the experiment, the curves overlap. Differences emerged only in the last week of experiments as the stem growth had decreased in the severe stress group and significantly differed from the groups of C and M (*p* = 0.0004). Indeed, severe stress treatments exhibited the lowest stem diameter growth (0.67 ± 0.05 mm), while the highest growth was obtained with control irrigation (1.04 ± 0.06 mm), followed by moderate stress (0.97 ± 0.07 mm).

### 2.3. Physiological Measures

#### 2.3.1. SPAD Index

The model that best fitted the SPAD index data was the complete model with irrigation, PS use, and MD as fixed factors and individuals as random factors. The three-way interactions between all the fixed factors had a significant effect on the SPAD model (df = 4, *p* < 0.001), indicating that the SPAD index changed over time in relation to both irrigation levels and the addition of PS. Compared to the control group, a decrease of 1.77 SPAD units has been caused by severe stress onset over the experiment (estimate = −1.77, SE = 0.50, t = −3.51, *p* < 0.001). Using PS at a low concentration in moderate stress conditions resulted in a 2.45 decrease in the SPAD index over time, compared to the reference treatment, i.e., the combination of control irrigation and PS at a high concentration (estimate = −2.45, SE = 0.71, t = −3.47, *p* < 0.001). No differences were observed compared to the reference in the absence of PS, over all irrigation levels and time periods (*p* > 0.1).

As for the random factors, the variance explained by the individuals was higher than the residual variance (Variance = 25.89 and 16.17, respectively), suggesting that the individual plants contribute substantially to the variation in SPAD measurements.

**Figure 4:**
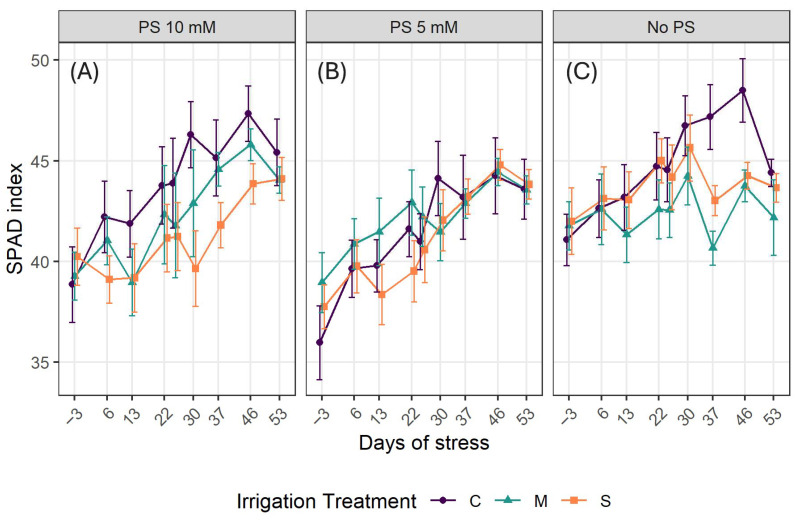
SPAD index evolution over time. Data are mean ± SE (n = 15). (**A**) PS use at 10 mM; (**B**) PS use at 5 mM; (**C**): No use of PS. C denotes control irrigation treatment (100% Field Capacity); M denotes moderate stress treatment (50% FC); S denotes severe stress treatment (25% FC).

The SPAD index time series (Figure 4) reveals an upward trend in PS-treated treatments (Figure 4A,B), although there is some fluctuation. Values without PS stagnate or decline slightly at certain points. Overall, chlorophyll content remains lower under water stress than under control irrigation. In the absence of PS use, the gap between stressed and well-watered plants widens over the course of the experiment, and the values decline. Conversely, in treatments involving biostimulants, the curves are more consistent and exhibit a clear trend for each irrigation treatment. Stressed plants supplemented with 10 mM potassium silicate SPAD index increased from approximately 40 at the beginning to 45–47 after 53 days of water stress onset, nearly matching the control value. In contrast, stressed plants without silicate remained 3–5 units lower (Figure 4A–C).

#### 2.3.2. Stomatal Conductance (*g*s)

To obtain the best model to fit the data on stomatal conductance, irrigation levels, and PS concentrations, days of stress were appointed as fixed factors and plant individuals as random factors. All the fixed factors had a significant influence on the data, but not in combination with each other (*p* < 0.05). The random factor of plant individuals had only a small influence on the data (variance = 0.0005), which was lower than the residual variance of 0.002. This indicates that differences among individual plants contribute minimally to the variation of *gs*.

Regarding the irrigation treatments, the stomatal conductance in moderate stress (M) did not differ significantly from well-watered treatments (C) (estimate = −0.011, SE = 0.006, t = −1.71, *p* = 0.08). Contrary to this, severe stress had significantly lower stomatal conductance than the control (estimate = −0.019, SE = 0.006, t = −2.96, *p* = 0.003). As for PS use, there was no significant difference between the two concentrations (*p* = 0.08). However, not using PS led to a 0.01 decrease in *gs* (estimate = −0.01, SE = 0.006, t = −2.44, *p* = 0.01). As time progressed, the model explained a decrease in *gs* of 0.003 (estimate = −0.003, SE = 0.001, t = −2.44, *p* = 0.01).

**Figure 5:**
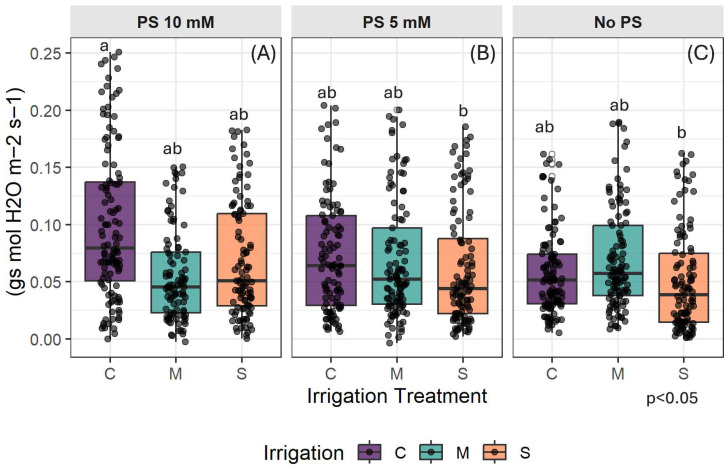
Distribution of *gs* as a function of PS concentration (**A**) PS at 10 mM: (**B**): PS at 5 mM; (**C**): No PS. C denotes control irrigation treatment (100% Field Capacity); M denotes moderate stress treatment (50% FC); S denotes severe stress treatment (25% FC). Data are mean ± SE (n = 15). The data represented are all the data throughout the experiment. Lowercase letters indicate significant differences between all treatments combined for *p* < 0.05.

The box plots in Figure 5 show that there is high variability in the response of stomatal conductance among seedlings. The control group (C) exhibits higher median values than the stressed groups, which suggests that stomatal conductance declines progressively with increasing water stress. The effect of PS is more pronounced in well-watered treatments and at a concentration of 10 mM, where the control group differs significantly from the M and S stressed treatments (Figure 5A). Furthermore, values were higher with 10 mM PS than with untreated plants (Figure 5C), particularly for the C and S treatments. Adding 5 mM PS does not appear to significantly increase the gas exchange rate compared to untreated plants.

### 2.4. Phytochemical Analysis

#### 2.4.1. Sugar Content in Leaves

ANOVA analysis concluded that the use of PS, irrigation levels, and their interaction had a significant influence on leaf sugar content at the end of the experiment (Supplementary Materials). Table 1 summarizes the concentration of each sugar class in each treatment, classified by PS concentration level. Overall, sugar concentration increases in stressful conditions regardless of whether PS is used. In treatments without PS, sucrose and glucose dominate, accounting for around 75% of the total sugar content. In PS-treated samples, sucrose accumulated significantly, accounting for about 60% of the total sugar content, followed by sorbitol. Without the use of PS, the maximum sugar levels were observed under moderate stress, then decreased under severe stress. With the use of PS, however, the levels were higher than with no use and remained the same between each treatment. For each sugar class, the concentrations of fructose and sorbitol increased in response to stress, an effect that was further intensified by the addition of PS. Glucose content remained stable across all irrigation treatments with 10 mM PS and without PS, but increased under stressful conditions with 5 mM PS. As can be seen from the total sugar content, *C. madagascariense* accumulates markedly higher levels of soluble sugars under water stress. Using PS amplifies this phenomenon even further.

#### 2.4.2. Secondary Metabolism

• Total phenol compounds (TPC)

**Figure 6:**
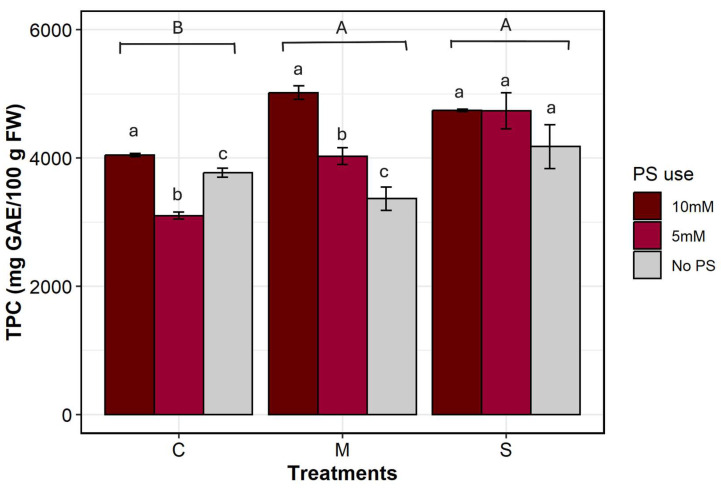
Total polyphenol content. C denotes control irrigation treatment (100% Field Capacity); M denotes moderate stress treatment (50% FC); S denotes severe stress treatment (25% FC). Data are mean ± SE (n = 9). Different lowercase letters indicate significant differences in PS use within the same irrigation treatment, and different uppercase letters indicate significant differences between irrigation treatments (*p* < 0.05).

Different levels of PS concentration and irrigation treatment, as well as their two-way interaction, have had a significant effect on the TPC (df = 2, *p* = 0.0009; df = 2, *p* = 0.0004; df = 4, *p* = 0.018, respectively). Indeed, TPC varied significantly across each irrigation × PS treatment. As can be seen in Figure 6, TPC shows a plausible increase under stress when PS was added. The highest value was observed in severe stress treatments, which increased from 4177.77 ± 340.80 to 4743.19 ± 20.32 mg _GAE_/100g FW. This was followed by moderate stress treatments, which increased from 3365.50 ± 184.16 to 5020.12 ± 105.56 mg _GAE_/100g FW. The lowest value was observed in the control group, which varied from 3770.22 ± 67.68 to 4047.19 ± 24.62 mg _GAE_/100g FW.

TPC was higher under stressful conditions than under optimal conditions. Under severe stress conditions, biosynthesis of polyphenols was so intense that adding PS had no effect; levels remained the same regardless of its use (S-Sbh-Sbl). Adding PS at a high concentration masked the effect of stress on TPC, which remained consistent across all irrigation treatments (Cbh, Mbh, Sbh). Moderate stress treatment was more responsive to the addition of PS at different concentrations than severe stress treatment. In the control treatments, the addition of PS at a high concentration increased the TPC by 7%, whereas at a low concentration, it decreased by 18%. Under moderate stress conditions, the addition of PS led to increases of 49% and 19%, respectively, at 10 mM and 5 mM concentrations. For severely stressed plants, TPC increased by a further 13% at both PS concentrations.

• Phenolic compounds

Four classes of polyphenols were detected from the HPLC: cinnamic acids, flavonols, benzoic acids, and catechins. The samples were particularly rich in flavonols and benzoic acids, containing more than 100 mg/100g_DW_ of each, but contained much less cinnamic acid (less than 10 mg/100g_Dw_). Overall, phenolic levels remained similar between irrigation treatments within the same PS concentration, with a few exceptions. With PS regardless, the content of cinnamic acids in Mbl and Mbh did not differ greatly from the control. This goes hand in hand with the highest content in other classes, such as flavonols. Benzoic acid content did not differ between treatments in all levels of PS concentration. The concentration remains similar regardless of the different treatments (Table 2). As for flavonols, only the irrigation treatment influenced their concentration (df = 2, *p* = 0.008). However, with the use of PS at high concentration, flavonols contents increase with stress. Similarly, only the use of PS influenced the concentration of catechins (df = 2, *p* = 0.02). Indeed, the concentrations are maintained similarly even under stress conditions. Regarding the cinnamic acids, irrigation treatments and PS concentration interaction significantly affect the concentrations (df = 4, *p* < 0.001). Without PS, the concentration remains similar for all irrigation treatments. However, the addition of PS increases cinnamic acids, especially under severe stress conditions, regardless of the concentration.

#### 2.4.3. Phytohormones

Four hormones were quantified: 2-cis-4-trans-abscisic acid (ABA), indole-3-acetic acid (IAA), indole-3-propionic acid (IPA), and indole-3-butyric acid (IBA). IBA had the highest concentration of all the hormones, while ABA had the lowest. There was no difference in IAA content across the treatments (*p* > 0.1). However, the contents of ABA, IPA, and IBA varied significantly between the different treatments.

Overall, the two-way interaction between irrigation levels and PS use had a significant effect on the level of ABA and IPA (*p* < 0.001; *p* = 0.013, respectively). As for IAA and IBA, only the irrigation treatments affected the data (*p* = 0.004 and *p* = 0.08, respectively).

**Figure 7:**
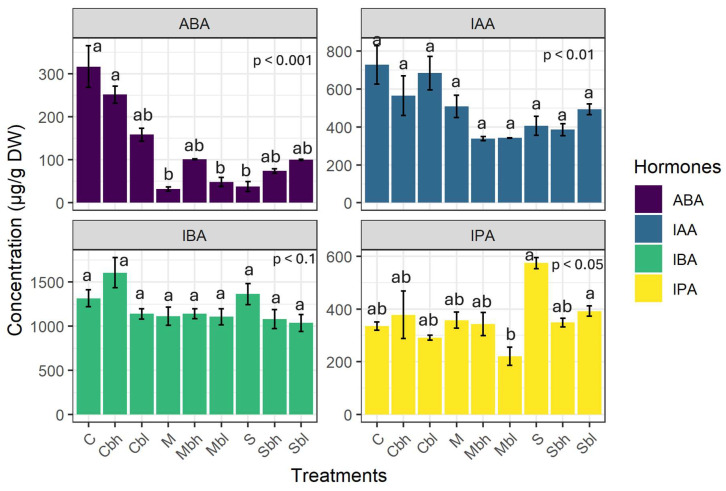
Leaf concentration of different phytohormones: 2-cis-4-trans-abscisic acid (ABA), indole-3-acetic acid (IAA), indole-3-propionic acid (IPA), and indole-3-butyric acid (IBA); Data are mean ± SE (n = 9). C, M, and S denote control irrigation treatment, moderate stress, and severe stress without the use of PS, respectively. Cbh, Mbh and Sbh treatments represent the control irrigation treatment and moderate and severe stress, respectively, with a PS concentration of 10 mM. Cbl, Mbl, and Sbl denote the control irrigation treatment, moderate and severe stress, respectively, with PS at 5 mM. Different lowercase letters in each panel indicate significant differences between treatments.

Figure 7 shows the leaf hormone profiles for each treatment. As can be seen, IBA is the most abundant hormone in all treatments, with levels ranging from 1000 to 1600 µg/g.

However, Cbh stands out with the highest concentration found. IBA levels were twofold, threefold, and tenfold higher than those of IAA, IPA, and ABA, respectively. ABA had the lowest level, which was particularly low in water stress treatments (from 23 to 359 µg/g). IAA levels were not statistically different in all treatments, varying from 310 to 970 µg/g. IPA levels increased markedly in severe stress conditions, ranging from 138 to 564 µg/g. In the case of ABA and IPA, the use of PS only had a slight effect on the hormone profiles, as the concentrations were similar in the treated and untreated samples.

## 3. Discussion

### 3.1. Morphological Attributes

Drought stress induces a reduction in plant morphological growth as an adaptation to harsh conditions. In the present study, there was a reduction in stem diameter and height in water-deficient conditions, with no significant counteraction observed when PS was used, even though we compensated for the potential loss of PS due to leakage and immobilization problems by using foliar spraying. To date, few studies have tested the efficacy of silicon-based products in improving the drought stress resistance of wild tree species. A study on wild tropical tree seedlings in Panama evidenced the same results regarding the lack of improvement in growth following the addition of silicon to the substrate in drought conditions [25] and an improvement of the latter in optimal watering conditions [24]. Furthermore, foliar spraying watermelons with a silicon-based biostimulant did not increase their fresh biomass weight [31] whereas in gerbera (*Gerbera jamsonii*) [32] and in cannabis (*Cannabis sativa*) [33], silicon significantly improves the height growth.

Limited water availability reduces cell turgidity and cell wall elasticity, which affects cell enlargement and growth. Under these conditions, photosynthesis cannot proceed normally due to harm to photosynthetic machinery and impaired gas exchange, coupled with the overaccumulation of ROS [10]. However, the mode of action of silicon focuses more on structural and functional modification [17]. Indeed, silicon is deposited onto the cell walls, forming a second layer of silica that prevents water loss through transpiration. This improves the plant’s water status and protects the cell from dehydration and oxidation [15]. Moreover, it enhances water extraction from the soil by the roots and water potential by regulating the relevant genes, as well as enhancing biochemical and physiological defences. The combination of these mechanisms leads to the maintenance of vital processes such as photosynthesis, nutrient absorption and osmotic balancing [15,17,32,34]. It has been found that, rather than promoting growth at reasonable concentration, excessive silicon application may cause plants to allocate more resources to stress tolerance and structural support than to height development [16]. Our results point to a strong functional and biochemical defence mechanism against stress in seedlings.

According to our results, the level of irrigation has a greater influence on the stem diameter than the use of PS. Indeed, stem thickening results from intense cellular division, inducing the production of secondary vascular tissue bundles from the cambium meristem. Cell division is more influenced by auxin hormones, but is less sensitive to water availability compared to cell expansion [10,35]. Moreover, plant water status is the main regulator of stem diameter fluctuations [36]. The stem diameter is also a function of the plant’s height and correlates with drought resistance. Taller plants tend to have wider vessels, making them more susceptible to embolisms and drought stress [37]. Reducing the height and width of the stem is either part of the plant’s own strategy for resisting drought or the result of reallocating resources to vital processes.

### 3.2. Physiological Mechanisms

Non-destructive and user-friendly methods, such as SPAD and porometer, that provide an instant diagnosis of the state of seedlings were chosen to assess their physiological responses, as these results will then be complemented by the investigation of the biochemical compounds through analyses. The measurement of leaf chlorophyll content using SPAD units is a widely adopted and established method as a scientifically validated tool for the assessment of leaf chlorophyll content and overall plant health. Compared to chemical extraction, the SPAD meter provides a quick and non-destructive field measurement and is particularly suitable for studies where destructive sampling is limited. However, its application remains limited in studies on forest species. For the studied species, no previous research has specifically investigated this physiological parameter in relation to water stress, whereas SPAD index remains one of the most reliable non-destructive methods to assess the nitrogen content directly correlated to chlorophyll content in the leaves, also in forest species [38]. Previous research has also demonstrated a strong correlation between SPAD readings and laboratory-determined chlorophyll content in threatened tropical forest species [39].

Our results showed that the SPAD index exhibited a sawtooth evolution over the course of the experiment and was significantly affected by the addition of the biostimulant, the levels of irrigation and the interaction between these two factors (Figure 4). The SPAD index can fluctuate depending on the growth stage of the plants and is significantly affected by water stress and nitrogen. Indeed, the SPAD index tends to be lower under stress conditions than under optimal conditions. Several studies reported similar results [34,40,41,42]. When under water stress, plants adopt a water-saving strategy to prevent water loss through stomatal adjustment. This leads to impaired transpiration and reduced carbon dioxide assimilation. Consequently, plants cannot process as much sunlight as usual and reduce chlorophyll content to absorb less light and prevent overheating and cell damage [43]. Moreover, low water availability in the soil reduces the uptake of nutrients such as nitrogen and magnesium, which are involved in the biosynthesis of chlorophyll pigments [44,45,46]. When combined with stomata closure, which results in an insufficient CO_2_ supply, the processes of photosynthesis become impaired, and the synthesis of chlorophyll is lessened [47]. Enzymes involved in chlorophyll synthesis are reduced, and chlorophyllase activity increases under water stress [48]. In addition, water stress triggers the production of large quantities of reactive oxygen species. If the plant’s antioxidant defences are unable to counterbalance these, oxidative stress is caused. This leads to the destruction of chlorophyll pigments due to photo-oxidation, which causes a decrease in leaf chlorophyll content when there is a shortage of water [42,47]. Using PS, especially at a high concentration, increases the SPAD index (Figure 4). Several researchers have found that adding potassium silicate increases chlorophyll content under stressful conditions [12,15]. This compound has many benefits for plant resistance to water stress due to silicon and potassium complementary roles. Potassium acts as an osmotic regulator, maintaining the normal functioning of stomata to ensure photosynthetic CO_2_ fixation. It also helps to maintain turgor pressure within cells, thereby regulating conditions within chloroplasts. Potassium also plays a role in activating enzymes and generating H^+^-ATPase, which is important for controlling photosynthetic rates [49]. Meanwhile, silicon is deposited onto the apoplasts of cell walls, creating a barrier made of silica cuticle that maintains the cell hydration status by limiting transpiration as well as protecting the photosynthetic apparatus. This increases the relative water content of the leaves by limiting transpiration [12,15]. Silicon enhances photosynthesis by increasing the concentration of chlorophyll and the activity of the RuBisCO and PEP carboxylase enzymes involved in CO_2_ fixation [50]. Furthermore, potassium silicate can counteract the destruction of the chlorophyll pigment caused by oxygen radicals by enhancing antioxidant activity and activating several antioxidant enzymes, including superoxide dismutase (SOD), catalase (CAT), and peroxidase (POD) [12,51]. Therefore, potassium silicate did enhance *C. madagascariense* tolerance to water stress. According to [32], the ability of a plant to synthesize and protect photosynthetic pigments in response to water restriction can indicate its tolerance to water stress.

Stomatal conductance (*gs*) is an important parameter for understanding the fluxes of CO_2_ and H_2_O between plants and their environment. This parameter responds quickly to stress and can be used as an early indicator of stress in plants, including *C. madagascariense,* as concluded in a previous study. It provides information on how plants respond to stressors and is therefore a key indicator of behavioral patterns in response to stress. Stomatal conductance exhibited significant variability within treatments despite the homogeneity of measurement methods. This is an intrinsic problem of stomatal conductance as it depends on vapor pressure deficit, temperature, light intensity, leaf position, and the measurement point on the leaf. High inter-individual variability can occur due to the seedlings’ differing adaptation strategies in response to soil water deficit at various stages of the experiment. The same situation was observed in a coniferous tree –the silver fir [52]. Furthermore, the stomatal conductance behaviour of different leaves of the same plant may vary depending on the local water status, hydraulic conductivity, and environmental conditions. Stomatal density, size, and clustering are important factors that can influence stomata response to stress on the magnitude and the rapidity of stomatal response [53]. In our study, water stress conditions reduced *gs,* while application of PS improved this parameter (Figure 5). The application of PS benefited not only plants with restricted irrigation, but also those with adequate watering. The same result was obtained in chinese licorice (*Glycyrrhiza uralensis*) [54], in common wheat (*Triticum aestivum*) [55], and in sorghum (*Sorghum bicolor*) [56,57]. Silicon plays a role in intensifying root water absorption and transport, enabling plants to retain more water and maintain their water status [54]. The addition of silicon has been shown to enhance stomata regulation, ensuring stomata aperture even during stressful conditions. Furthermore, the literature well-documented the role of potassium in osmotic control of guard cells, thereby regulating stomatal function and behavior [58]. In constrained environmental conditions, plants adjust the degree of stomatal aperture as an immediate adaptation to reduce water loss, which is correlated with a significant reduction in *gs*. However, in the case of more persistent conditions, plants undergo structural changes, such as altering the size of stomata or their density [59]. The reduction of stomata size is more common in plants, being responsible for *gs* decline during water shortages [60]. A study in sugarcane (*Saccharum officinarum*) suggested that silicon enhances the ability of plants to open their stomata, even under stressful conditions, indicating its role in regulating stomatal aperture [59]. This enables the plants to maintain gas exchange, ensuring CO_2_ uptake and the continuation of photosynthesis processes. Furthermore, the addition of silicon through spray and/or soil application had a more significant impact on maintaining stomatal conductance and regulating water status, although the effect was more pronounced with foliar spraying at high concentrations [61].

### 3.3. Biochemical Responses

Polyphenols are important components that help plants defend themselves against abiotic stress. They account for a large proportion of the antioxidant defence against oxidative stress by neutralizing ROS [62]. Total phenol content increased with stress (Figure 6), which is consistent with other studies on different tree species [63,64]. Drought stress modulates the biosynthesis pathway of phenolic compounds, triggering an increase in levels that are crucial for defence against the negative effects of stress [62]. Moreover, this effect was intensified by the addition of PS at high concentrations. Some authors reported that a higher concentration of silicon improved the biosynthesis of polyphenols in daisy (*Bellis perennis*) [12], in chestnut (*Castanea sativa*) [22], and in tomato (*Solanum lycopersicum*) [65]. It has been demonstrated that higher TPC is an indicator of stress tolerance and adaptation in plants [22]. The biosynthesis of polyphenols is triggered by stress. Through different signalling pathways, such as the overproduction of reactive oxygen species (ROS) and/or abscisic acid (ABA) synthesis [66], the genes that encode phenylalanine ammonia-lyase (PAL), the enzyme involved in the phenylpropanoid pathway, are activated and overexpressed. PAL channels the transfer of carbon from primary metabolism into secondary metabolism: the synthesis of phenolic compounds [67,68]. PAL catalyzes the conversion of phenylalanine into trans-cinnamic acids. This is followed by the action of cinnamate 4-hydroxylase (C4H) and 4-coumarate: CoA ligase (C4L) enzymes, which produce the precursor molecule p-coumaryl-CoA for the biosynthesis of various secondary metabolites [69]. Furthermore, providing plants with silicon combined with potassium has been proven to induce changes in the expression of genes involved in the phenylpropanoid pathway, which encodes enzymes involved in phenol synthesis, such as PAL. This enhances their activity, resulting in intense production of phenolic compounds. Particularly, silicon induced a myriad of reactions through the phenylpropanoid pathway to produce and accumulate defence molecules such as polyphenols [12,70,71].

The phenolic profiles (Table 1) showed that the plants produced more flavonols and benzoic acids, whereas cinnamic acids were only present in trace amounts. Catechin content remains stable between treatments. Indeed, to maintain ROS homeostasis, plants implement enzymatic and non-enzymatic pathways for ROS suppression. Flavonols represent the non-enzymatic pathway plays an important role in how plants cope with stressful conditions, given their particularly potent antioxidant capacity. They can also control root development and auxin transport [72,73]. Flavonol levels were particularly high under moderate stress conditions with the addition of PS. This suggests a quicker and more intense conversion of cinnamic acids into naringenin, the precursor of flavonoids that turns into kaempferol, the origin of flavonols, with the priming effect of PS [74,75,76]. Benzoic acid assures the function of photosynthesis and transpiration regulation as well as ion transport and uptake [77]. Catechins have a high antioxidant capacity and regulate genes involved in adapting to water shortages, including those activating the phenylpropanoid pathway. [78]. Although cinnamic acid is present in very low concentrations, its content increases with stress, regardless of PS use, especially under severe stress conditions. This was confirmed in alfalfa (*Medicago sativa*) [79]. In the phenylpropanoid pathway, cinnamic acids are the first intermediates, which are less stable and are immediately transformed into downstream metabolites that are more stable for storage [76]. The presence of low levels of cinnamic acids, together with high levels of downstream phenolic compounds such as flavonols, benzoic acids, and catechins, most likely indicates high PAL activity and rapid downstream metabolism rather than accumulation of early intermediates. *C. madagascariense* may be naturally rich in flavonols and benzoic acids, as its content remains similar regardless of treatments. Flavonols play different roles in human health, such as providing antioxidant, anti-inflammatory, and antiviral properties [80].

Plants are classified as primary producers because they can produce the components necessary for their growth, such as sugar, independently through photosynthesis. In this study, the content of soluble sugars increased under stressful conditions and when PS was used (Table 1). Such results were confirmed by several authors [12,22,81]. Nevertheless, without the use of PS, sugar levels were higher under moderate stress than under severe stress, as observed in soybeans [82]. The effect of stress on photosynthesis inhibition was harsher under severe drought, redirecting the metabolism to sugar degradation rather than synthesis. This opposite trend in the presence and absence of PS suggests that PS can influence sugar metabolism by promoting the degradation of sugar into simpler and rather than its accumulation in a more stable form. Soluble sugars are widely recognised as osmotic regulators that contribute to stabilizing cell membranes and regeneration, as well as providing energy for vital processes. One of the adaptation mechanisms deployed by plants, especially in the event of more intense water shortage, is to increase sugar content to adjust osmosis and maintain cell water balance, thereby protecting cells from dehydration and protein denaturation [83,84]. Silicon increases the production of photosynthetic pigments, thereby enabling photosynthesis. It also mitigates oxidative stress and protects macromolecules [20]. Consequently, sugar production continued despite underuse, which led to reduced plant growth and the accumulation of sugars.

All the samples, especially those from stress treatments, exhibited significant sucrose accumulation, which increased with the use of PS. Numerous studies on different plants, such as chinese licorice (*Glycyrrhiza uralensis*) and soybean (*Glycine max*), have found this accumulation of sucrose [81,85]. Two enzymes are involved in sucrose synthesis: Sucrose phosphate synthase (SPS) and sucrose synthase (SuSy). The latter acts in both synthesis and breakdown. In our study, drought did not inhibit sucrose synthesis and the activity of these enzymes. It is possible that silicon intervened in boosting the actions of SuSy and SPS by upregulating the relevant genes [81]. The lower fructose and glucose content indicates that sucrose production is prioritised over its degradation. Sucrose can stabilise cell membranes and proteins, protecting them from desiccation by forming a glass-like substance within the cells. It is also easily mobilised and transported, making it a better osmoregulator [86]. In fact, it has been proven that a high accumulation of sucrose indicates enhanced resistance to stress in woody plants [87,88].

Sorbitol, also known as sugar alcohol, derives from common sugar differentiated by the skeleton with multiple hydroxyl groups [89]. In our experiment, we found that the sorbitol content increased in the leaves. This accumulation of sorbitol during stress has also been observed in *Malus* [88] and *Prunus* genera [90]. Alongside the common sugar, this sugar alcohol participates in adjusting osmotic potential and acts as an ROS scavenger, as well as slowing down ROS production [91]. Stress tolerance has been proven to be correlated with an accumulation of sorbitol [92].

Hormones intervene at every stage of a plant’s life, modulating the functioning of biological processes. While each hormone class plays a distinct role, they also interact with each other. In the event of drought stress, they act as chemical messengers, transmitting the stress signal and helping plants to modulate their response [17]. In this study, we only quantified all the hormones at the end of the experiment rather than monitoring them at different stages of the experiment, as this required destructive samplings of plant leaves. This could have affected how the plants functioned, making subsequent measurements of the other parameters studied incomparable. At this stage of the study, the objective was to gain an understanding of the cumulative effect of the entire water stress period and the use of PS on hormonal balance. However, investigating the dynamics of the hormones throughout the different stages of the experiment could provide further insight into hormone regulation during each stage. This will be explored in a future study. In the present study, the hormone profiled (Figure 7) showed a predominance of IBA, a hormone in the auxin family, which is undoubtedly linked to its role in regulating root formation and development. It is involved in modifying root architecture by promoting the growth of lateral and adventitious roots, root hair elongation [93,94]. The IAA concentration was 30 times higher in *C. madagascariense* than in grape vine *Vitis vinifera* leaves, where the maximum concentration reported was 50 µg/g [95]. It has been found that high levels of IAA could indicate a better resistance to drought as the latter improves antioxidant defence, upregulates relevant gene expression, and regulates other hormone levels [96]. The accumulation of IAA, the main hormone in the auxin family responsible for growth and development, could be a growth biomarker [97]. IPA was also detected as it plays complementary roles with IBA in the modulation of lateral root growth [98]. Contrary to our expectations, the ABA content of the leaves was found to be the lowest of all the hormones and decreased even further under water-deficit stress compared to optimal conditions. Moreover, ABA decreased with stress application with the addition of silicon, probably due to the alleviation of drought stress in plants [99]. Some research has found that ABA levels decrease over a prolonged period of drought stress. It was demonstrated that ABA production follows a biphasic trend (peaking-type). In the early stage of drought, when water potential drops, ABA levels continually increase until they reach a peak corresponding to when potential is close to turgor loss, after which they decline. This phenomenon was observed in angiosperm species such as slender mulga (*Acacia aptaneura*) and california laurel (*Umbellularia californica*), as well as most gymnosperm species, *Callitris rhomboidea *(oyster bay pine) [100,101]. This phenomenon is linked to a high xylem resistance toward embolism and anisohydric stomatal regulation in drought conditions [102]. This could be a plausible explanation, given that ABA was only quantified at the end of the two-month experiment. However, further research is required to confirm this biphasic trend in *C. madagascariense*. The initial ABA levels before stress onset should be established, and ABA levels and leaf water potential should be monitored throughout the duration of the stress to understand how ABA secretion evolves as leaf potential decreases, and to see the overall trend in ABA secretion.

Elevated auxin concentration can improve drought tolerance mechanisms by enhancing antioxidant activity and modulating root system architecture by stimulating root lateral development [103]. On the one hand, it is possible that ABA could follow a biphasic pattern throughout the stressful period. On the other hand, it is also possible that potassium silicate can modulate the hormonal pathway and crosstalk. Potassium, by itself, plays multiple roles in the response to drought stress by influencing auxin signaling mechanisms and maintaining plant turgor pressure. The addition of potassium has been shown to improve IAA content under both optimal and stressful conditions [104]. The interaction between these two hormones improves water uptake. Considering the role of IAA in modifying root system architecture, potassium optimizes the plant’s water status while reducing ABA concentration to enable gas exchange and CO_2_ assimilation through controlled stomatal closure [105]. Our results suggest that stomatal conductance improves with the use of PS in both stressful and optimal conditions (Figure 5). This is consistent with low levels of ABA, which could also be linked [106].

## 4. Materials and Methods

### 4.1. Experiment Set-Up and Plant Materials

The experiment was carried out in a greenhouse at the Ecole Supérieure des Sciences Agronomiques (University of Antananarivo, Analamanga, Madagascar) in November and December 2024. A total of 135 two-year-old *C. madagascariense* plants, grown in 1.413 L plastic nursery bags, were used for the experiment. The plants were sourced from Avotr’Ala nursery in Moramanga (Alaotra Mangoro, Madagascar). The voucher number for the species used is MNHN-P-P00048611 (Muséum national d’Histoire naturelle, Paris). All the seedlings benefited from a one-month preconditioning period involving daily irrigation near to water retention capacity and NPK fertilisation (11:22:16) at a rate of 12 g before the start of the irrigation treatments. A randomised block design was adopted for the experiment. The relative humidity and temperature inside the greenhouse were monitored throughout the experiment using the humidity and temperature sensor EasyLog EL-GFX-2 (Lascar Electronics, Salisbury, UK).

### 4.2. Treatments

This experiment tested two factors: the irrigation level and the concentration of the potassium silicate solution. In our previous study, adding potassium silicate at 10 mM to the soil did not conclusively demonstrate its efficacy for the *C. madagascariense*. The literature suggests that foliar application alone or in combination with soil application gives better results in improving plant resilience and tolerance to hydric stress [22,107]. Based on [22], two concentrations of PS (K_2_ Si O_3_, Cerrus ©, Cerrus S.A.S, Uboldo, Italy) were tested: a low concentration of 5 mM and a higher concentration of 10 mM and a combined foliar spray and soil application was adopted. Fifty milliliters of the solution were added to the soil, and the same amount was sprayed onto the aerial parts of the plants. The application was carried out one week before the drought onset. This product provides 23.5% of silicon in the form of SiO_2_ and 10.49% of potassium in the form of K_2_O.

Three irrigation levels were chosen for the daily watering of the seedlings: 100% was used as the control irrigation level (C), while 50% and 25% were the stress treatment levels (M and S, respectively). These percentages are reported to the water content at the field capacity point. The field capacity point of the substrate was determined in accordance with the protocol described by [108]. Three pots filled with the substrate were watered abundantly until the water began to leak from the pots. The pots were then immediately covered with plastic sheets to prevent evaporation while the excess water drained away. The pots were placed in a dark, fresh location and left to drain for two days. After this time, soil samples were taken from each pot, weighed, and placed in an oven at 105 °C until completely dry, i.e., until the weight remained constant. The difference in weight between the soil at the field capacity point and the oven-dried soil gives the amount of moisture at the field capacity point (344.65 ± 20.41 g). The pots were irrigated to field capacity one day before the onset of the stress treatment. The weight of each pot was recorded daily. The difference between the daily weight and the reference weight determined the amount to be given to each pot. The trial consisted of nine treatments, each involving five plants and replicated three times, arranged in a randomized block design (Table 3).

**Table 3:** Experimental treatments.

	Control Irrigation (100%)	Moderate Stress (50%)	Severe Stress (25%)
Without potassium silicate (PS)	C	M	S
PS at 10 mM	Cbh	Mbh	Sbh
PS at 5 mM	Cbl	Mbl	Sbl

### 4.3. Plant Monitoring and Measurements

The height of the plants from the collar to the terminal bud of the main stem, and the stem diameter at one centimetre above the collar, were measured fortnightly using a conventional rigid tape measure and digital callipers, respectively.

The chlorophyll content was assessed, through the SPAD index, every 4 days on two fully developed leaves with the Arborcheck © ArbCm-01 (Hansatech Instruments Ltd., Pentney, UK). As for stomatal conductance (*g*s), measurements were taken using a LI-600 dynamic porometer (LI-COR Environmental, Lincoln, NE, USA) at four-day intervals on the upper and lower fully developed leaves, which were fully illuminated at zenith.

### 4.4. Metabolite Quantification

Phenolics, as well as different sugars and phytohormones content, were determined from the leaves collected at the end of the experiment.

#### 4.4.1. Total Phenol Content and Phenolic Compounds

The extraction was performed using 5 g of fresh leaves and 50 mL of an extraction solvent consisting of a mixture of methanol, hydrochloric acid (37%) and distilled water [109]. The total phenol compounds (TPC) were determined using the Folin–Ciocalteu reagent [110]. The absorbance was read on a UV/Vis spectrophotometer (1600-PC, VWR International Srl, Milan, Italy) at a wavelength of 760 nm.

Two different chromatographic methods were performed using an Agilent 1200 High-Performance Liquid Chromatograph connected to an Agilent UV-Vis diode array detector (Agilent Technologies, Santa Clara, CA, USA) for phenolic identification and quantification. A Phenomenex Kinetex C18 column (4.6 × 150 mm, 5 μm, Phenomenex, Torrance, CA, USA) was used to separate the polyphenols [111].

#### 4.4.2. Sugar Quantification

Two grams of dried leaves from each treatment were ground using an electric grinder (Black & Decker, New Britain, CT, USA). Approximately 2 g of dried leaves were weighed from each sample. 50 mL of an extraction solvent consisting of 90% ethanol was added to the sample. The sample was then centrifuged at 3000 rpm for 15 min to ensure the liquid extract was free of solid deposits. The samples were characterised by chromatographic analysis. A Sphere Clone—NH2 column (4.6 × 250 mm, 5 μm) was used for sugar separation (sucrose, fructose, glucose and sorbitol). The concentration was then determined using a standard calibration curve [112].

#### 4.4.3. Phytohormone Quantification

The protocol, as described by [113], was applied with slight modifications. 500 mg of dried leaf samples were used for the analysis. 4 mL of an extraction solvent composed of 65% methanol, 10% ultrapure water, and 25% HCl (1 M) was added to the samples and left to macerate in the dark for one hour. During the maceration process, the content of each sample vial was mixed using a vortex mixer for approximately one minute every 20 min. Then, the samples were centrifuged at 3000 rpm for 15 min to produce a clear extract, free from solid particles. The extracts were stored in a refrigerated unit before being transferred to HPLC vials and injected into the column for chromatographic analysis. A C18 Kinetex (150 × 4.6 mm × 5 µm) was used for the stationary phase, and a solution of H_3_PO_4_ and acetonitrile was used for the mobile phase. Each analysis lasted for 50 min with 5 min of conditions and a flow rate of 0.3 mL/min. Four phytohormones were identified during this procedure: indole-3-acetic acid (IAA), 2-cis-4-trans-abscisic acid (ABA), indole-3-propionic acid (IPA) and indole-3-butyric acid (IBA). The concentration was then determined using a standard calibration curve [114].

#### 4.4.4. Statistical Analysis

For continuous measurements such as height, SPAD index, and stomatal conductance, linear mixed models (nlme package) were used to analyse the data to account for the variation between each block at different measurement dates, which could potentially affect the results [115]. The model includes irrigation modalities, PS concentrations, and measurement dates (MD) as fixed effects and experimental blocks and plants id as random effects. The step function helped in the selection of the best model based on the Akaike Information Criterion. Chi-square tests at a significance level of 0.05 were performed to compare each model and understand the effect of the fixed factors and their interactions, to better fit the model. Before interpreting the models, the assumptions of normality and homoscedasticity were verified using Q-Q plots and residuals vs. fitted values plots. The summary command gave all the outputs of the model both on fixed (variance estimate, standard error and *p* value) and random effects (variance). An analysis of variance (ANOVA) was performed on the linear mixed model to facilitate interpretation of the model. Where treatments had significant differences, a pairwise comparison was made using the emmeans function (emmeans package). As for punctual data, such as phytochemical analysis data, a simple ANOVA was performed on Gaussian distributed data to compare the effects of different treatments, followed by Tukey’s HSD test at significance levels of 0.05 and 0.1. All statistical analyses, graphs and charts were made using R Software version 2024.04.2 (R Core Team).

## 5. Conclusions

In conclusion, drought stress altered the functional responses of *C. madagascariense*, resulting in reduced growth. However, the saplings were able to deploy resistance and tolerance mechanisms to mitigate the negative effects. This study aimed to assess the potential of using potassium silicate to increase *C. madagascariense*’s resistance to water stress. This species demonstrates drought tolerance through the risky anisohydric-like strategy of maintaining a higher gas exchange rate with the assistance of PS. Furthermore, the species possesses a strong antioxidant defense and osmoprotection, which was further improved with the addition of PS. Consequently, the seedlings were able to withstand stress and improve their biochemical compound content, establishing the species as a medicinal plant. Overall, combining foliar spraying with an in-soil addition increased the effectiveness of PS in eliciting positive responses in *C. madagascariense*, even at low concentrations. However, a high concentration may divert resources towards chemical defence rather than growth. Previous findings from an unpublished study, considered alongside the present study, suggest that foliar spraying combined with soil incorporation greatly improves the efficacy of potassium silicate on *C. madagascariense*, even at a low concentration of 5 mM. The two tested concentrations showed only minor differences across most parameters, suggesting that the effective range clearly lies between 5 and 10 mM. Although higher concentration may offer slightly greater improvements, the lower concentration still provides substantial benefits, particularly when factors such as environmental sustainability and cost-effectiveness are taken into account. It is possible that the hormone profiles obtained showed that the species had high resilience to water-deficient situations. Nevertheless, further investigation is needed to characterize the physiological defense mechanisms underlying the stress response. The use of highly soluble silicon is therefore confirmed to be effective in producing vigorous seedlings under water-saving conditions, with the goal of *C. madagascariense* restoration in the forest in the event of potential drought. Future studies could evaluate the transcriptomic and metabolomic aspects underlying the responses observed in this preliminary study, as well as the potential of locally sourced, silicon-rich materials to promote more sustainable circular resource management practices.

## Figures and Tables

**Table 1:** Sugar content per PS concentration.

Treatments	Fructose g/100g_Dw_	Glucose g/100g_Dw_	Sorbitol g/100g_Dw_	Sucrose g/100g_Dw_	Total g/100g_Dw_
NO PS					
C	0.060 ± 0.005 b**	2.233 ± 0.807 n.s.	0.771 ± 0.187 b*	1.180± 0.070 b**	4.244 ±1.066 b**
M	0.078 ± 0.012 b**	3.647± 0.853 n.s.	1.668 ± 0.617 ab*	2.898 ± 0.294 a**	8.291 ± 0.679 a**
S	0.232 ± 0.062 a**	2.694 ± 0.026 n.s.	1.213 ± 0.059 a*	1.750 ± 0.539 ab**	5.889 ± 0.448 ab**
PS 5 mM					
Cbl	0.073 ± 0.002 b**	0.347 ± 0.088 c**	1.822 ± 0.096 a**	5.773 ± 1.687 a**	8.014 ± 1.678 a**
Mbl	0.566 ± 0.125 a**	1.342 ± 0.144 b**	1.135 ± 0.160 b**	4.940 ± 0.622 a**	7.984 ± 0.726 a**
Sbl	0.108 ± 0.011 b**	3.776 ± 0.639 a**	n.d.	6.353 ± 1.191 a**	10.236 ± 0.552 a**
PS 10 mM					
Cbh	0.118 ± 0.011 b*	0.278 ± 0.033 a**	1.222 ± 0.225 a**	4.580 ± 0.820 a**	6.198 ± 1.034 a**
Mbh	0.157 ± 0.026 b*	0.259 ± 0.028 a**	1.989 ± 0.056 a**	5.454 ± 1.114 a**	7.860 ± 1.064 a**
Sbh	0.562 ± 0.250 a*	0.442 ± 0.208 a**	2.206 ± 0.868 a**	4.773 ± 0.028 a**	7.983 ± 0.546 a**

Values are expressed as mean ± standard deviation (n = 9), n.d.: not detected. C denotes control irrigation treatment (100% Field Capacity); M denotes moderate stress treatment (50% FC); S denotes severe stress treatment (25% FC). Data are mean ± SE (n = 15). Post hoc tests were performed for each sugar at each PS concentration. Bold lowercase letters indicate significant differences between treatments for each sugar class with (**) *p* < 0.01, (*) *p* < 0.05, no asterisk 0.05 ≤ *p* ≤ 0.1, n.s.: non-significant.

**Table 2:** Content of the different polyphenol classes in leaves by PS concentration.

Treatments	Cinnamic Acids mg/100g_Dw_	Flavonols mg/100g_Dw_	Benzoic Acids mg/100g_Dw_	Catechins mg/100g_Dw_
No PS				
C	2.54 ± 0.30 a**	141.13 ± 7.24 a**	160.63 ± 48.31 n.s.	87.36 ± 17.65 a**
M	3.46 ±1.42 a**	178.88 ± 51.60 a**	135.70 ± 54.27 n.s.	64.05 ± 17.21 a**
S	3.12 ± 0.99 a**	190.42 ± 42.68 a**	111.10 ± 26.16 n.s.	70.32 ± 25.36 a**
PS 5 mM				
Cbl	3.36 ± 0.32 b**	160.48 ± 39.69 a**	112.55 ± 18.21 n.s.	46.91 ± 13.33 a**
Mbl	3.42 ± 0.50 b**	200.51 ± 43.05 a**	159.55 ± 35.44 n.s.	54.91 ± 9.29 a**
Sbl	10.82 ± 0.92 a**	175.62 ± 18.44 a**	146.96 ± 12.14 n.s.	65.74 ± 20.40 a**
PS 10 mM				
Cbh	4.91 ± 0.91 b**	135.84 ± 24.20 b**	118.82 ± 3.31 n.s.	49.71 ± 7.01 a**
Mbh	2.70 ± 0.81 b**	222.65 ± 2.37 a**	151.08 ± 25.89 n.s.	48.24 ± 5.86 a**
Sbh	8.25 ± 0.64 a**	180.73 ± 32.50 ab**	158.38 ± 56.03 n.s.	66.43 ± 10.16 a**

Values are expressed as mean ± standard deviation (n = 9). C denotes control irrigation treatment (100% Field Capacity); M denotes moderate stress treatment (50% FC); S denotes severe stress treatment (25% FC). Post hoc tests were performed for each phenolic class at each PS concentration. Bold lowercase letters indicate significant differences between irrigation treatments for each phenolic class with (**) *p* < 0.01 and no asterisk 0.05 ≤ *p* ≤ 0.1. n.s.: non-significant.

## Data Availability

The data will be made available upon request from the corresponding author. The data are not publicly available due to privacy.

## Supplementary Materials

The following supporting information can be downloaded at: https://www.mdpi.com/article/10.3390/plants14243760/s1.

## Abbreviations

The following abbreviations are used in this manuscript:

PS	Potassium silicate
C	Treatment irrigated at 100% of FC
Cbh	Treatment irrigated at 100% of FC with 10 mM of potassium silicate
Cbl	Treatment irrigated at 100% of FC with 5 mM of potassium silicate
M	Treatment irrigated at 50% of FC
Mbh	Treatment irrigated at 50% of FC with 10 mM of potassium silicate
Mbl	Treatment irrigated at 50% of FC with 5 mM of potassium silicate
S	Treatment irrigated at 25% of FC
Sbh	Treatment irrigated at 25% of FC with 10 mM of potassium silicate
Sbl	Treatment irrigated at 25% of FC with 5 mM of potassium silicate
SPAD	Soil–Plant Analysis Development
PAL	Phenylalanine ammonia-lyase
SuSy	Sucrose Synthase
SPS	Sucrose phosphate synthase
IAA	Indole-3-acetic acid
ABA	2-cis-4-trans-abscisic acid
IPA	Indole-3-propionic acid
IBA	Indole-3-butyric acid
