# Supplementary material for: Advances in Potassium Silicate-Induced Drought Tolerance in Tropical Tree Seedlings: Effects on Morphological Traits, Physiological Responses, and Biochemical Regulation
Source: Plants (Basel). 2025 Dec 10;14(24):3760. doi: 10.3390/plants14243760 (PMC12736938; doi:10.3390/plants14243760)
Supplement: Supplementary file 1 [file plants-14-03760-s001.zip › plants-3988219-supplementary.pdf]

DIAMETER MODEL SUMMARY

Linear mixed model fit by REML. t-tests use Satterthwaite's method [lmerModLmerTest]

Formula: diam ~ irr * date_s + (1 | id)
Control: lmerControl(optimizer = "bobyqa")

REML criterion at convergence: 914.8

Scaled residuals:

Min	1Q	Median	3Q	Max
-3.9240	-0.5251	-0.0305	0.5058	6.4345

Random effects:

Groups	Name	Variance	Std.Dev.
id	(Intercept)	1.84055	1.3567
Residual		0.08769	0.2961

Number of obs: 670, groups: id, 135

Fixed effects:

	Estimate	Std. Error	df	t value	Pr(>|t|)
(Intercept)	7.39392	0.20324	131.99570	36.380	< 2e-16 ***
irrM	-0.27248	0.28740	131.94809	-0.948	0.34482
irrS	-0.08663	0.28740	131.95499	-0.301	0.76357
date_s	0.35438	0.02002	531.99516	17.705	< 2e-16 ***
irrM:date_s	-0.09058	0.02812	531.96650	-3.221	0.00136 **
irrS:date_s	-0.16037	0.02814	531.96914	-5.699	2e-08 ***

HEIGHT MODEL SUMMARY

Linear mixed model fit by REML. t-tests use Satterthwaite's method ['lmerModLmerTest']

Formula: hei ~ irr + date_s + (1 | id) + irr:date_s

REML criterion at convergence: 2194.8

Scaled residuals:

Min	1Q	Median	3Q	Max
-6.9633	-0.4131	0.0136	0.3723	6.1142

Random effects:

Groups	Name	Variance	Std.Dev.
id	(Intercept)	1.3245	1.1509
Residual		0.9921	0.9961

Number of obs: 675, groups: id, 135

Fixed effects:

	Estimate	Std. Error	df	t value	Pr(>|t|)
(Intercept)	1.96978	0.18396	132.00000	10.707	< 2e-16 ***
irrM	-0.90667	0.26016	132.00000	-3.485	0.000668 ***
irrS	-0.91111	0.26016	132.00000	-3.502	0.000630 ***
date_s	1.36707	0.06645	537.00000	20.572	< 2e-16 ***
irrM:date_s	-0.64861	0.09398	537.00000	-6.902	1.45e-11 ***
irrS:date_s	-0.73319	0.09398	537.00000	- 7.802	3.21e-14 ***

Signif. codes: 0 ‘***’ 0.001 ‘**’ 0.01 ‘*’ 0.05 ‘.’ 0.1 ‘ ’ 1

SPAD MODEL SUMMARY

Linear mixed model fit by REML. t-tests use Satterthwaite's method ['lmerModLmerTest']
Formula: spad ~ 1 + irr * ps * date_s + (1 | id)

REML criterion at convergence: 6912.1

Scaled residuals:

Min	1Q	Median	3Q	Max
-6.4426	-0.5314	0.0436	0.6016	3.5175

Random effects:

Groups	Name	Variance	Std.Dev.
id	(Intercept)	25.89	5.088
Residual		16.17	4.021

Number of obs: 1172, groups: id, 134

Fixed effects:

	Estimate	Std. Error	df	t value	Pr(>|t|)
(Intercept)	43.8707	1.3586	124.0993	32.290	< 2e-16 ***
irrM	-1.5526	1.9236	124.6554	-0.807	0.421136
irrS	-3.6251	1.9238	124.7028	-1.884	0.061842 .
psbl	-2.4113	1.9214	124.0993	-1.255	0.211856
psn	1.4924	1.9217	124.1669	0.777	0.438871
date_s	2.1679	0.3463	1028.7430	6.261	5.61e-10 ***
irrM:psbl	2.5308	2.7196	124.5094	0.931	0.353862
irrS:psbl	2.5023	2.7201	124.6078	0.920	0.359382
irrM:psn	-0.3712	2.7198	124.5433	-0.136	0.891647
irrS:psn	2.3633	2.7460	124.9052	0.861	0.391073
irrM:date_s	-0.1605	0.5023	1031.5848	-0.320	0.749392
irrS:date_s	-1.7749	0.5062	1030.8711	-3.506	0.000474 ***
psbl:date_s	0.1347	0.4897	1028.7430	0.275	0.783323
psn:date_s	0.5021	0.4925	1028.9226	1.019	0.308206
irrM:psbl:date_s	-2.4505	0.7057	1030.8819	-3.472	0.000537 ***
irrS:psbl:date_s	0.7091	0.7110	1030.6036	0.997	0.318838
irrM:psn:date_s	-1.1015	0.7076	1030.9567	-1.557	0.119855
irrS:psn:date_s	-0.1622	0.7310	1031.7717	-0.222	0.824415

Signif. codes: 0 '***' 0.001 '**' 0.01 '*' 0.05 '.' 0.1 ' ' 1

STOMATAL CONDUCTANCE MODEL SUMMARY
Linear mixed model fit by REML. t-tests use Satterthwaite's method ['lmerModLmerTest']
Formula: gs ~ 1 + irr + ps + date_s + (1 | id)

REML criterion at convergence: -2893.1

Scaled residuals:

Min	1Q	Median	3Q	Max
-2.2639	-0.6761	-0.2072	0.5106	3.8738

Random effects:

Groups	Name	Variance	Std.Dev.
id	(Intercept)	0.0005614	0.02369
Residual		0.0025968	0.05096

Number of obs: 984, groups: id, 125

Fixed effects:

	Estimate	Std. Error	df	t value	Pr(>|t|)
(Intercept)	0.091372	0.005867	119.384688	15.575	< 2e-16 ***
irrM	-0.011192	0.006519	119.581397	-1.717	0.08859 .
irrS	-0.019324	0.006524	119.909090	-2.962	0.00369 **
psbl	-0.011354	0.006608	120.341453	-1.718	0.08834 .
psn	-0.015937	0.006510	119.185577	-2.448	0.01581 *
date_s	-0.003997	0.001633	860.076485	-2.448	0.01457 *

Signif. codes: 0 '***' 0.001 '**' 0.01 '*' 0.05 '.' 0.1 ' ' 1

ANOVA FRUCTOSE

	Df	Sum Sq	Mean Sq	F value	Pr(>F)
irr	2	5.7931	2.8965	62.974	7.474e-09 ***
ps	2	2.5416	1.2708	27.628	3.265e-06 ***
irr:ps	4	7.9931	1.9983	43.445	5.175e-09 ***
Residuals	18	0.8279	0.0460		

Signif. codes: 0 '***' 0.001 '**' 0.01 '*' 0.05 '.' 0.1 ' ' 1

ANOVA GLUCOSE

	Df	Sum Sq	Mean Sq	F value	Pr(>F)
irr	2	4.6160	2.3080	35.390	5.789e-07 ***
ps	2	21.9550	10.9775	168.327	2.235e-12 ***
irr:ps	4	4.8341	1.2085	18.531	3.420e-06 ***
Residuals	18	1.1739	0.0652		

Signif. codes: 0 '***' 0.001 '**' 0.01 '*' 0.05 '.' 0.1 ' ' 1

SORBITOL

	Df	Sum Sq	Mean Sq	F value	Pr(>F)
irr	2	0.41728	0.20864	3.7044	0.047634 *
ps	2	0.80188	0.40094	7.1187	0.006146 **
irr:ps	3	1.29155	0.43052	7.6438	0.002164 **
Residuals	16	0.90116	0.05632		

Signif. codes: 0 '***' 0.001 '**' 0.01 '*' 0.05 '.' 0.1 ' ' 1

SUCROSE

	Df	Sum Sq	Mean Sq	F value	Pr(>F)
irr	2	0.41728	0.20864	3.7044	0.047634 *
ps	2	0.80188	0.40094	7.1187	0.006146 **
irr:ps	3	1.29155	0.43052	7.6438	0.002164 **
Residuals	16	0.90116	0.05632		

Signif. codes: 0 '***' 0.001 '**' 0.01 '*' 0.05 '.' 0.1 ' ' 1

TOTAL SUGAR

	Df	Sum Sq	Mean Sq	F value	Pr(>F)
irr	2	21.391	10.6955	12.1146	0.0004645 ***
ps	2	30.560	15.2800	17.3074	6.419e-05 ***
irr:ps	4	19.429	4.8574	5.5018	0.0044954 **
Residuals	18	15.892	0.8829		

Signif. codes: 0 '***' 0.001 '**' 0.01 '*' 0.05 '.' 0.1 ' ' 1

TOTAL PHENOLIC CONTENT

	Df	Sum Sq	Mean Sq	F value	Pr(>F)	
irr	2	3606679	1803340	13.9566	0.0004639	***
ps	2	3082629	1541314	11.9287	0.0009459	***
irr:ps	4	2188955	547239	4.2352	0.0187649	*
Residuals	14	1808950	129211			

Signif. codes: 0 ‘***’ 0.001 ‘**’ 0.01 ‘*’ 0.05 ‘.’ 0.1 ‘ ’ 1

CINNAMIC ACIDS

	Df	Sum Sq	Mean Sq	F value	Pr(>F)	
irr	2	96.658	48.329	70.512	3.050e-09	***
ps	2	40.200	20.100	29.326	2.171e-06	***
irr:ps	4	61.895	15.474	22.576	8.227e-07	***
Residuals	18	12.337	0.685			

FLAVONOLS

	Df	Sum Sq	Mean Sq	F value	Pr(>F)	
irr	2	14033.4	7016.7	6.3446	0.008215	**
ps	2	506.8	253.4	0.2291	0.797515	
irr:ps	4	3715.6	928.9	0.8399	0.517756	
Residuals	18	19906.8	1105.9			

BENZOIC ACID

	Df	Sum Sq	Mean Sq	F value	Pr(>F)
irr	2	1480.5	740.27	0.5784	0.5709
ps	2	218.4	109.18	0.0853	0.9186
irr:ps	4	8410.3	2102.58	1.6428	0.2070
Residuals	18	23037.4	1279.85		

CATECHINS

	Df	Sum Sq	Mean Sq	F value	Pr(>F)	
irr	2	623.4	311.72	1.3247	0.29059	
ps	2	2078.2	1039.08	4.4158	0.02752	*
irr:ps	4	1398.5	349.61	1.4858	0.24807	
Residuals	18	4235.6	235.31			
